# The prognostic value of peripheral blood inflammatory markers in patients with HER2-positive metastatic breast cancer treated with pyrotinib

**DOI:** 10.3389/fonc.2026.1812069

**Published:** 2026-05-08

**Authors:** Wenqi Zhou, Jing Wu, Peng Zhang, Jing Xiong, Yi Yang, Xiaohua Zeng, Wei Li

**Affiliations:** 1 Department of Breast Center, Chongqing Key Laboratory for Intelligent Oncology in Breast Cancer (iCQBC), Chongqing University Cancer Hospital, Chongqing, China; 2 Gastrointestinal, Thyroid and Breast Surgery Department, Chongqing University Qianjiang Hospital, Chongqing, China

**Keywords:** HER2 positive breast cancer, LDH, NLR, prognosis, pyrotinib

## Abstract

**Background:**

Different peripheral blood markers have been reported to be involved in different stages of breast cancer development and metastasis. However, the association between NLR, LDH and prognosis of pyrotinib in HER2-positive metastatic breast cancer (mBC) remains unknown.

**Methods:**

We collected the clinical data and blood test results of 113 patients with HER2-positive mBC who were treated with pyrotinib at Chongqing University Cancer Hospital. The clinical data and blood test indexes were collected, and the ROC curve determined the optimal cut-off value. Kaplan-Meier survival curve and Cox regression model was used to analyze the effect of different levels of NLR and LDH before T-DM1 treatment on the survival of patients.

**Results:**

The optimal cut-off value of NLR and LDH was 2.44 (p=0.027) and 216.5U/L (p=0.033), respectively. With a median follow-up of 49.0 months (95%CI: 44.2-53.8), the median PFS and OS were 12.0 months (95%CI: 8.9-15.1) and 36.0 months (95%CI: 30.12-41.88), respectively. High NLR was associated with only a trend toward worse PFS (low-NLR vs high-NLR: 14.0 months vs 11.0 months, p=0.095). While patients with low LDH had a significantly longer PFS (19.0 months) than patients with high LDH (10.0 months, p<0.001). Higher NLR or LDH were significantly correlated with shorter median OS (low-NLR vs high-NLR: not reach (NR) vs 33.0 months, p=0.021; low-LDH vs high-LDH: NR vs. 30.0 months, p=0.002). Multivariable models showed that LDH was the only covariate independently associated with worse PFS (p=0.002), while high LDH (p=0.021), high NLR (p=0.007) and postmenopause (p=0.034) were independently associated with worse OS.

**Conclusions:**

Serum NLR and LDH were prognostic risk factors for metastatic HER2 positive breast cancer patients treated with pyrotinib. Elevated NLR and LDH were associated with poor PFS and OS. Future data are needed to validate and update our results.

## Introduction

Human epidermal growth factor receptor 2 (HER2) is an important therapeutic target and HER2-positive breast cancer accounts for 20%-30% of all breast cancers (1). HER2-positive breast cancer is highly malignant, prone to recurrence and metastasis, leading to a poor prognosis. Anti-HER2 targeted therapy has brought a landmark change for HER2-positive breast cancer patients and significantly improved the prognosis of these patients. For patients with metastatic breast cancer, the standard first-line treatment includes taxane chemotherapy combining with trastuzumab and pertuzumab (2, 3). In the second- and later-line settings, currently available targeted drugs mainly include antibody-drug conjugate (ADC) drugs such as trastuzumab deruxtecan (4, 5), trastuzumab emtansine (6, 7) and small molecule tyrosine kinase inhibitors (TKIs) such as pyrotinib (8–10), tucatinib (11) and lapatinib (12).

Pyrotinib is a new type of irreversible TKI targeting HER1, HER2, and HER4. Previous studies have shown good tolerability and promising antitumor activity in patients with heavily pretreated HER2-positive metastatic breast cancer (mBC) (13–15). A phase II study demonstrated that pyrotinib plus capecitabine was superior to lapatinib plus capecitabine in patients with HER2-positive metastatic breast cancer who had failed trastuzumab. Pyrotinib plus capecitabine had a significantly higher objective response rate (ORR) (78.5% vs 57.1%) and a significantly longer median progression-free survival (PFS)(18.1 months vs 7.0 months) compared with lapatinib plus capecitabin (8). The phase III PHOEBE (10) clinical study further confirmed that pyrotinib combined with capecitabine could significantly prolong the median PFS (12.5 months vs 6.8 months) and improve the ORR (67% vs 52%) of HER2-positive mBC patients. Based on these studies, pyrotinib plus capecitabine is recommended as an effective second - or later-line treatment option for patients with advanced breast cancer who have failed trastuzumab. However, there are still some patients who do not respond to this regimen. Therefore, it is necessary and meaningful to find biomarkers that can predict the effect of pyrotinib.

Several studies have confirmed that inflammation process plays a crucial role in the occurrence, recurrence and metastasis of tumors, including breast cancer (16–18). It was reported that many inflammatory cells, including neutrophils, lymphocytes, monocytes and their corresponding ratios, such as the neutrophil-to-lymphocyte ratio (NLR), lymphocyte-monocyte ratio (LMR), have be used to evaluate the treatment response and predict the prognosis (19–23). Furthermore, the nomogram based on multiple inflammatory markers could also be tailored to assess the prognosis of breast cancer patients (24). In addition, serum lactate dehydrogenase (LDH) has been proved to be associated with the prognosis of various tumors (25–28). LDH is involved in the anerobic glycolysis process of tumor growth and proliferation, and it mediates immune escape by inhibiting CD8+T lymphocytes and natural killing activation (29). Higher LDH values were noted in the triple-negative breast cancer, positive lymph nodes, and lymphovascular invasion patients, and might indicate poor outcomes (28). However, the role of these biomarkers in HER2-positive mBC patients treated with pyrotinib remains unclear. Therefore, we explored the potential role of NLR and LDH as prognostic biomarker in HER2-positive mBC patients treated with pyrotinib.

## Materials and methods

### Patient eligibility

This retrospective study included 113 HER2-positive mBC patients who underwent pyrotinib treatment at Chongqing University Cancer Hospital between January 2020 and December 2022. The inclusion criteria were as follows: (1) breast cancer with unresectable locally recurrent or metastatic disease diagnosed by cytology or histology; (2) pathologically confirmed HER2 positive (defined as IHC3+ or FISH+); (3) patients without brain metastases were required to have received previous anti-HER2 treatment with trastuzumab ± pertuzumab; (4) availability of baseline (pre-treatment) absolute counts of neutrophils, lymphocytes and lactate dehydrogenase (LDH) in peripheral blood (5) available information on the date of disease progression according to Response Evaluation Criteria In Solid Tumors (RECIST) 1.1 criteria. The main exclusion criteria were: (1) patients with blood diseases, acute and chronic infections, renal diseases, and other diseases or factors that may affect blood indicators; (2) patients had received colony-stimulating factor or blood products within 2 weeks before treatment with pyrotinib. Eligible patients received pyrotinib (400mg once daily) combined with single-agent chemotherapy (capecitabine or vinorelbine according to previous regimens). Dose reductions of pyrotinib were allowed in a stepwise manner, from 400 mg to 320 mg, then to 240 mg based on tolerance. This research complies with the ethical principles for medical research involving human subjects adopted in the Declaration of Helsinki and was approved by the medical ethics committee of the Chongqing University Cancer Hospital (No. CZLS2023271).

Peripheral blood collection and measurement.

The blood sample were obtained within 1 week before pyrotinib treatment. After fasting for 12 hours, 2.0 mL peripheral venous blood was collected in the morning. Sysmex XN9000 hematology analyzer was used for blood routine examination. We collected blood-related inflammatory markers, including absolute neutrophil count (ANC), absolute lymphocyte count (ALC), and LDH. In addition, the corresponding ratios containing neutrophil-to-lymphocyte ratio (NLR) were analyzed.

### Observation indicators

The efficacy was evaluated every 2 to 3 cycles of treatment by computerized tomography (CT). PFS was calculated from pyrotinib treatment start to the date of radiological or clinical documentation of progressive disease (PD), last follow-up or death, whichever occurred first (censored at last follow-up for patients alive and without PD). OS was calculated from treatment start to the date of death or last follow-up (censored at last follow-up for patients alive).

### Statistical analysis

All analyzes were conducted using SPSS version 25.0 (IBM Corp., Armonk, NY, USA). Continuous data were expressed as mean ± standard deviation or the median(range). The appropriate cut-off values of NLR and LDH for predicting OS were calculated by using the receiver operating characteristic (ROC) curve and the area under the curve (AUC) calculated by the Youden index. The correlation between the biomarkers and clinicopathological variables was tested by using chi-square test, Fisher’s exact test or continuity correction as appropriate. Kaplan-Meier curve and log-rank test for PFS and OS were applied for each group. The Cox proportional hazards model was used for univariate and multivariate analysis of survival data to obtain hazard ratio (HR) and 95% confidence interval (95%CI). A p value < 0.05 was considered statistically significant.

## Results

A total of 113 mBC patients were eligible for analysis. The median age was 53 years old. More than half of them were postmenopausal (75.2%), 70.8% of them were previously treated with trastuzumab, three quarters of the patients had visceral metastases, and one third had brain metastases, most patients (86.7%) received two or more lines of treatment before. Baseline characteristics of patients are summarized in **Table 1**.

**Table 1:** Baseline characteristics of advanced breast cancer patients.

Variable	Number (%)
Age (years)	
<50	33 (29.2)
≥50	80 (70.8)
ECOG PS	
0	96 (85.0)
1 and 2	17 (15.0)
Menstrual status	
premenopause	28 (24.8)
postmenopause	85 (75.2)
MBC status	
De novo	63 (55.8)
Recurrent	50 (44.2)
Previous target treatment	
Trastuzumab	80 (70.8)
Trastuzumab and pertuzumab	14 (12.4)
Trastuzumab and ADC	5 (4.4)
None	14 (12.4)
Visceral metastasis	
Yes	85 (75.2)
No	28 (24.8)
Brian metastasis	
Yes	34 (30.1)
No	79 (69.9)
Treatment line	
<2	15 (13.3)
≥2	98 (86.7)
HER2 status	
HER2(IHC2+) FISH(+)	48 (42.5)
HER2(IHC3+)	65 (57.5)
HR status	
Positive	50 (44.2)
Negative NLR	63 (55.8)
≥2.44	74 (65.5)
<2.44	39(34.5)
LDH	
≥216.5	42 (37.2)
<216.5	54 (47.8)

**Figure 1:**
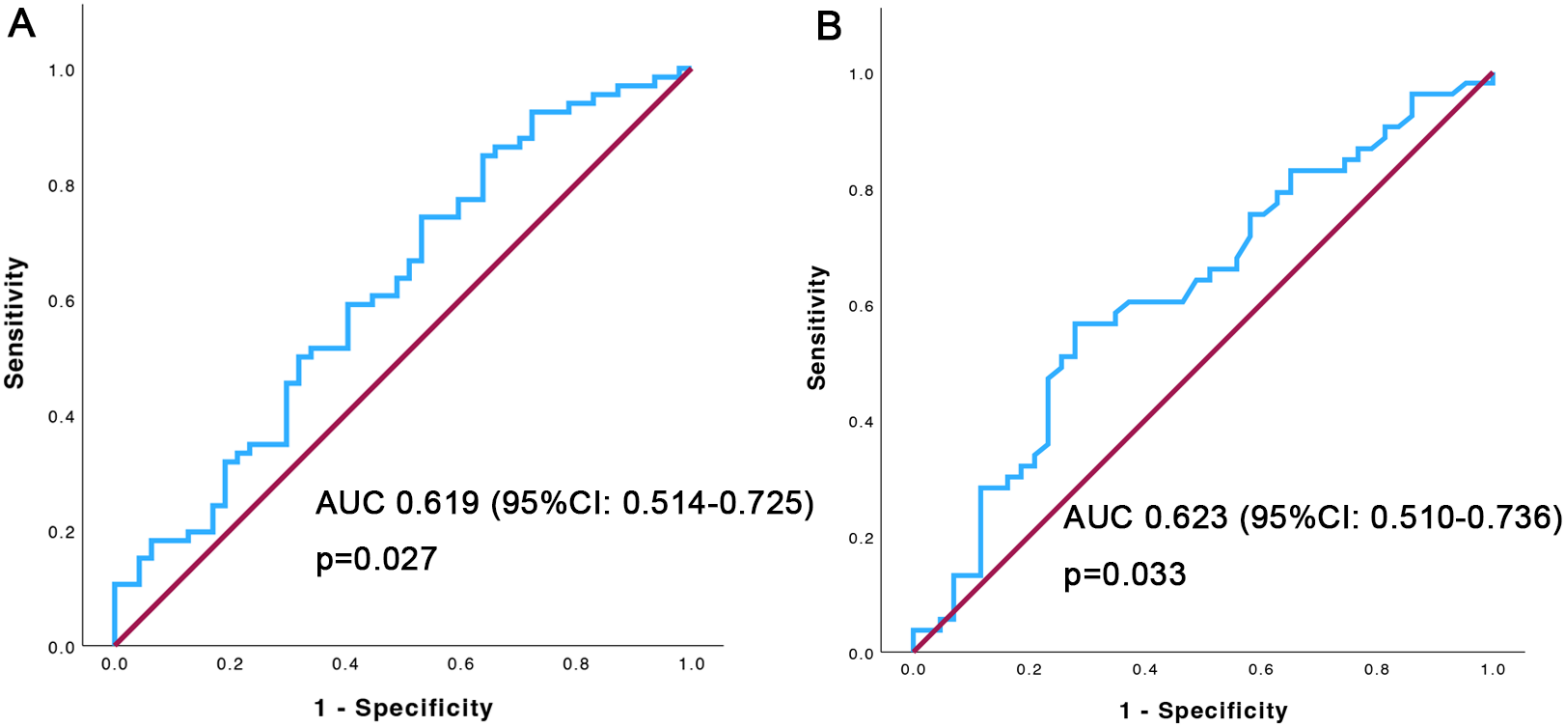
ROC curve of NLR **(A)** and LDH **(B)** for predicting overall survival.

The mean NLR value was 3.22 ± 1.72(range: 0.49 -10.87). According to ROC analysis, the NLR value of 2.44 had a sensitivity of 74.2% and specificity of 46.8%, and the area under the curve was 0.619 (95%CI, 0.514-0.725), p=0.027. An NLR> 2.44 was defined as high NLR, and NLR ≤ 2.44 was defined as low. Only 96 patients had their pretreatment LDH values collected. The mean LDH was 239.25 ± 116.66 U/L (range: 115.00-731.00), the cut-off value of LDH was 216.5U/L, with a sensitivity of 56.6% and specificity of 71.2%, and the area under the curve was 0.623 (95%CI, 0.510-0.736), p=0.033. The ROC curve was shown in **Figure 1**.

Neither NLR nor LDH seemed to have a significant association with the clinicopathological characteristics of patients, except that in the high LDH group, the proportion of patients over 50 years old seemed to be higher. The relationship between clinical characteristics and the parameters is shown in **Table 2**.

**Table 2:** Associations Between Parameters and Clinicopathological characteristics.

Variable	NLR			LDH		
	Low	High	P-value	Low	High	P-value
Age (years)						
<50	9 (23.1)	24 (32.4)	0.298	20 (37.0)	7 (16.7)	0.028
≥50	30 (76.9)	50 (67.6)		34 (63.0)	35 (83.3)	
ECOG PS						
0	30 (76.9)	66 (89.2)	0.083	44 (84.6)	37 (90.2)	0.376
1 and 2	9 (23.1)	8 (10.8)		10 (15.4)	5 (9.8)	
Menstrual status						
premenopause	6 (15.4)	22 (29.7)	0.093	17 (31.5)	6 (14.2)	0.050
postmenopause	33 (84.6)	52 (70.3)		37 (68.5)	36 (85.7)	
MBC status						
De novo	13 (33.3)	14 (18.9)	0.088	11 (20.4)	15 (35.7)	0.093
Recurrent	26 (66.7)	60 (81.1)		43 (79.6)	27 (64.3)	
Visceral metastasis						
Yes	29 (74.4)	56 (75.7)	0.878	39 (72.2)	31 (73.8)	0.862
No	10 (25.6)	18 (24.3)		15 (27.8)	11 (26.2)	
Brian metastasis						
Yes	11 (28.2)	23 (31.1)	0.751	16 (29.6)	11 (26.2)	0.710
No	28 (71.8)	51 (68.9)		38 (70.4)	31 (73.8)	
Metastasis site						
1	13 (33.3)	24 (32.4)	0.923	23 (42.6)	11 (26.2)	0.096
≥1	26 (66.7)	50 (67.6)		31 (57.4)	31 (73.81)	
Treatment line						
<2	5 (12.8)	10 (13.5)	0.918	5 (9.3)	5 (11.9)	0.933^a^
≥2	34 (87.2)	64 (86.5)		49 (90.7)	37 (88.1)	
HER2 status						
HER2(IHC2+) FISH(+)	14 (35.9)	34 (45.9)	0.304	21 (38.9)	18 (42.9)	0.695
HER2(IHC3+)	25 (64.1)	40 (54.1)		33 (61.1)	24 (57.1)	
HR status						
Positive	20 (51.3)	30 (40.5)	0.274	23 (42.6)	21 (50.0)	0.470
Negative	19 (48.7)	44(59.5)		31 (57.4)	21 (50.0)	

^a^Chi-squared by continuity correction.

**Figure 2:**
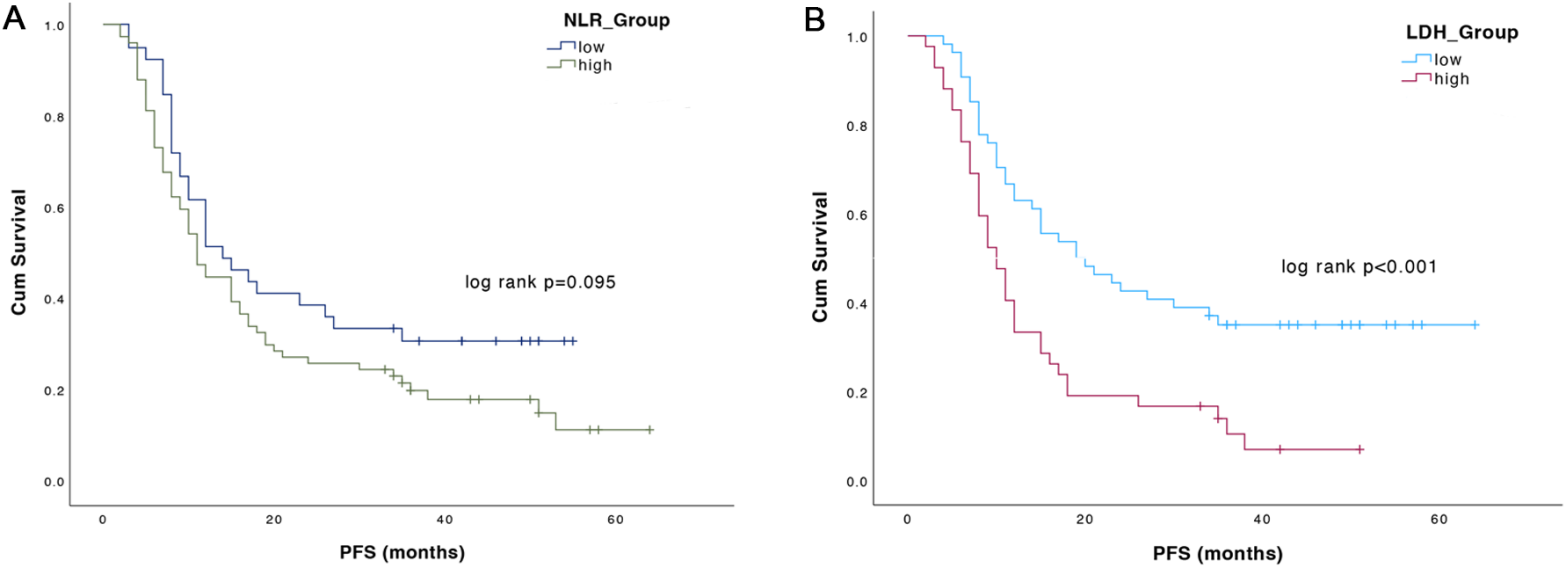
Progression free survival (PFS) according to NLR **(A)** and LDH **(B)** values.

With a median follow-up of 49.0 months (95%CI: 44.2-53.8), a total of 89 progression events were detected, with a median PFS of 12.0 months (95%CI: 8.9-15.1). High NLR was associated with only a trend toward worse PFS. Median PFS was 14.0 months (95%CI:7.9-20.1) for patients with low NLR compared with 11.0 months (95%CI: 8.7-13.3) for patients with high NLR (p=0.095) (**Figure 2A**). By contrast, patients with low LDH had a significantly longer PFS of 19.0 months (95%CI: 9.4-28.6) than patients with high LDH (median PFS 10.0 months, 95%CI: 7.6-12.4; p<0.001) **Figure 2B**).

**Figure 3:**
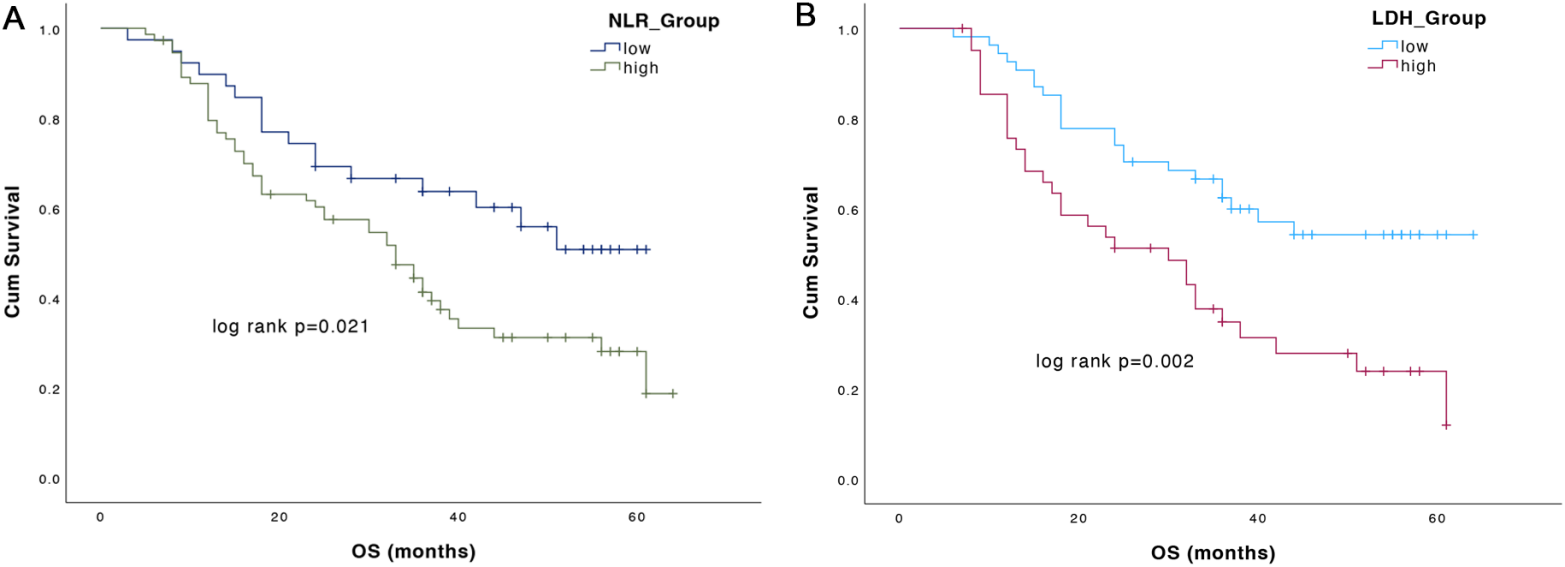
Overall survival (OS) according to NLR **(A)** and LDH **(B)** values.

During the study follow-up, 66 death events occurred, with a median OS of 36.0 months in the study population (95%CI: 30.12-41.88). The Kaplan-Meier analysis showed significant differences in OS between patients with different NLR and LDH. Higher NLR or LDH were associated with worse OS. Regarding NLR, median OS was 33.0 months (95%CI:24.11-41.89) in NLR high group, while it was not reach (NR) in NLR low group (p=0.021) (**Figure 3A**). As for LDH group, OS was significantly longer in patients with low baseline LDH as compared to high LDH (NR vs. 30.0 months, 95%CI: 17.0-43.0, p=0.002) (**Figure 3B**).

### Univariate and multivariate analyzes

In univariate analysis, factors associated with worse PSF were a higher LDH (p=0.001), and more metastatic sites (p=0.032). Visceral metastasis may serve as a potential negative prognostic biomarker (p=0.053). Multivariable models including the above 3 factors showed that LDH was the only covariate independently associated with worse PFS (p=0.002) (**Table 3**).

**Table 3:** Univariate and multivariable analysis of PFS.

Variable	Univariate		Multivariate	
	HR (95% CI)	P-value	HR (95% CI)	P-value
NLR (high vs. low)	1.45 (0.92-0.228)	0.106	2.14 (1.32-3.46)	
LDH (high vs. low)	2.18(1.37-3.47)	0.001		0.002
Visceral metastasis (yes vs. no)	1.65(0.99-2.75)	0.053	1.45 (0.73-2.89)	0.286
Age (≥50 vs. <50)	1.27 (0.79-2.03)	0.321		
Menopause (post vs. pre)	1.44(0.88-2.37)			
Brain metastasis (yes vs. no)	1.32 (0.84-2.07)	0.222		
Recurrent vs. De novo	0.83(0.52-1.34)	0.444		
Metastasis site (≥1 vs. 1)	1.67(1.05-2.66)	0.032	1.26 (0.66-2.40)	0.480
Treatment line (≥2 vs. <2)	1.02 (0.54-1.91)	0.963		
HER2 status (IHC3+ vs. IHC 2+ FISH+)	1.24(0.81-1.90)	0.317		
HR status (+ vs. -)	0.91(0.60-1.39)	0.664		

HR, hazards ratio; CI, confidence interval.

Regarding OS, higher LDH (p=0.003) and NLR (p=0.025), more metastatic sites (p=0.017), older age (p=0.025) and postmenopausal status (p=0.010) were associated with worse OS. Similarly, visceral metastasis may be a potential negative predictor of OS (p=0.061). Due to the interaction between age and menstrual status, only one of them could be included in the multivariable model. In combination with clinical practice, the menstrual status was selected for analysis. According to the results, high LDH (p=0.021), high NLR (p=0.007) and postmenopause (p=0.034) were independently associated with worse OS (**Table 4**).

**Table 4:** Univariate and multivariable analysis of OS.

Variable	Univariate		Multivariate	
	HR(95% CI)	P-value	HR(95% CI)	P-value
NLR (high vs. low)	1.88(1.08-3.28)	0.025	2.40 (1.28-4.53)	0.007
LDH (high vs. low)	2.31(1.34-3.98)	0.003	1.97 (1.11-3.48)	0.021
Visceral metastasis (yes vs. no)	1.77(0.97-3.23)	0.061	1.54 (0.73-3.28)	0.262
Age (≥50 vs. <50)	2.05(1.09-3.84)	0.025		
Menopause (post vs. pre)	2.51(1.24-5.07)	0.010	2.44 (1.07-5.58)	0.034
Brain metastasis (yes vs. no)	1.45 (0.866-2.433)	0.157		
Recurrent vs. De novo	0.99(0.56-1.77)	0.753		
Metastasis site (≥1 vs. 1)	1.99(1.13-3.51)	0.017	1.39 (0.67-2.87)	0.374
Treatment line (≥2 vs. <2)	0.89(0.44-1.81)	0.753		
HER2 status (IHC3+ vs. IHC 2+ FISH+)	1.23(0.75-2.02)	0.411		
HR status (+ vs. -)	0.97(0.60-1.59)	0.908		

HR, hazards ratio; CI, confidence interval.

## Discussion

In recent years, various serum biomarkers, such as inflammatory factors, circulating DNA and circulating tumor cell, have been identified as prognostic factors of malignant tumor. Indicators which can be obtained from routine blood biochemistry tests would be meaningful and cost-effective for patients to preliminarily assess the response to treatment and the prognosis of disease. NLR and LDH, as two routine diagnostic markers, which are commonly used in clinical practice, are proved to be associated with treatment response and prognosis of variety of malignant tumors, including breast cancer. However, the value of NLR and LDH in mBC treated with pyrotinib is still unclear.

In the present study, 113 mBC patients were included, most of them had visceral metastases, and received two or more lines of treatment. The median PFS was 12.0 months, which was consistent with the phase III PHOEBE trial (10). According to ROC curve, 2.44 and 216.5U/L was chosen to be the cut-off value of NLR and LDH respectively. Although the cut-off values for LDH and NLR were similar to some literatures, they varied a lot between different studies (20–23, 26, 27, 30, 31), some studies applied ROC curve analysis to calculate optimal cutoff values, while others adopted the normal value or upper limit of normal value as cutoff values. Factors related to the clinical variables, like tumor burden, different patient groups and races may also contribute to heterogeneity. Elevated NLR and LDH were associated with shorter PFS and OS, and both served as independent biomarkers of OS. However, only LDH was proved to be an independent prognostic indicator of PFS, while NLR only showed a certain statistical trend, which was different with previous research (20, 21, 31). The difference might be related to the small sample size or other factors such as different patient groups as well as different treatment.

The mechanisms underlying the correlation of NLR and tumor progression are widely explored. Chronic inflammatory is a significant mediator of cancer initiation and promotion (32). As a representative of systemic inflammatory, neutrophils are considered non-negligible in tumor growth and progression, especially in metastatic initiation (33). It works through releasing nitric oxide derivatives and reactive oxygen species (ROS) as well as through TGF-β induced signal pathway, which associated with high-grade malignancy (34, 35). As for lymphocytes, activated CD8+ cytotoxic T lymphocytes are the major component of immune response which destroy cancer cells, and the helper CD4+ T lymphocytes differentiate into type1 (Th1), which could facilitate immune response mediated by CD8+ T cells (36). The absolute neutrophil count is affected by various physical, pathological and physiological factors, while NLR is relatively stable in peripheral blood test (37), and it takes systemic inflammation as well as immune function into consideration, leading to a relatively comprehensive evaluation. NLR was proved to be a valuable predictive and prognostic biomarker in breast cancer patients treated with CDK4/6 inhibitor (21) and neoadjuvant chemotherapy (20). Notably, NLR could also predict the surgical advantage for *de novo* stage IV breast cancer patients (31).

LDH is a key rate-limiting enzyme in glycolysis, and it is involved in the anaerobic glycolysis process of tumor growth. It plays a critical role in promoting malignant tumor cell growth and inhibiting the body’s immune response against tumors by accelerating ATP production, tumor cell metabolism, and lactate production (38). In addition, LDH allows neoplastic cells to suppress and evade the immune system by inhibiting CD8+ T lymphocytes and natural killing (NK) activation, thus promotes resistance to chemo/radio/targeted therapy (29). High levels of LDH are associated with massive cell death, acute diseases, and tissue destruction caused by the cancerous growth (27). Like NLR, LDH can be detected in clinical practices, the prognostic value of LDH has been established in various tumor, including breast cancer (22, 26, 27, 30, 39).

Both NLR and LDH are associated with inflammation, tissue damage and immune response. Taken together, we have reasons to believe that NLR and LDH might be of usefulness in predicting response to therapy and prognosis. Although the value of NLR in predicting PFS in patients treated with pyrotinib was not significant, it still showed prognostic role for OS. Larger sample size may further determine the significance of this indicator.

It was a retrospective and single-center study, and was based on short-term analysis, which had inevitably limitations. The sample size of this study was limited, especially for subgroup analysis. Small sample size may bias our results, thus multi-center, large-sample prospective studies are needed to validate our findings and improve the reliability and generalizability of the research results. Besides, the lack of dynamic monitoring of LDH and NLR was another deficiency of this study. The NLR and LDH kinetics were proved to be an index of response to the treatment (28, 40), which need to be evaluated in prospective studies. Also, it should be noted that the patients included in this study were of different treatment lines, which may lead to inconsistent results. Furthermore, although we identified the independent prognostic value of NLR and LDH, it may change a lot due to many reasons, so combining them with other factors like menopausal status, clinical TNM stage, tumor burden and other immune parameters might be more practical in clinical use. Although there are limitations in this study, we still hope it could provide exploration value for future studies.

## Conclusions

In summary, elevated NLR (>2.44) and LDH (>216.5U/L) were associated with shorter PFS and OS in HER2-positive mBC patients treated with pyrotinib, both of them may serve as independent predictive factor for OS. However, due to the limited sample size and single center design, multi-center and prospective study with larger sample size are needed to further verify the value of NLR and LDH in patients treated with pytotinib.

## Data Availability

The raw data supporting the conclusions of this article will be made available by the authors, without undue reservation.
